# The posttraumatic growth of fathers of preterm infants: protocol for a qualitative study in China

**DOI:** 10.3389/fpsyt.2024.1444226

**Published:** 2024-08-30

**Authors:** Lina Yin, Yanli Liu, Kejimu Sunzi, Dandan Huang, Jing Huang, Liangmei Tang, Minghui Liu

**Affiliations:** Department of Pediatric, People’s Hospital of Deyang City, Sichuan, Deyang, China

**Keywords:** posttraumatic growth, fathers, preterm infants, qualitative, protocol

## Abstract

**Background:**

Prematurity presents a significant life crisis for families, often exceeding their expectations. Fathers of premature infants face the burden of multiple caregiving roles and undergo psychological changes. When confronted with such crises, individuals often engage in self-evaluation and may experience positive transformations. This study aims to employ a qualitative research methodology to explore the experiences of fathers of preterm infants.

**Materials and methods:**

A phenomenological approach design will be utilized, drawing upon semi-structured in-depth interviews informed by existing literature. Thematic analysis will be employed, adhering to the Consolidated Criteria for Reporting Qualitative Research (COREQ) guidelines. In-depth individual interviews, lasting 40-60 minutes, will be conducted with fathers of preterm infants to understand their experiences. The thematic analysis process will facilitate a comprehensive understanding of the factors contributing to post-traumatic growth among these fathers. This methodology provides a structured approach to investigating the experiences and influences on post-traumatic growth in fathers of preterm infants.

**Results:**

This study will highlight changes in post-traumatic growth among fathers of preterm infants.

**Discussion:**

Research on the post-traumatic growth (PTG) of fathers of preterm infants is crucial to understanding the unique challenges and psychological transformations they experience. This study aims to explore the factors contributing to PTG in these fathers and how cultural contexts in China influence this process. By elucidating these aspects, the findings can inform targeted interventions and support systems tailored to the needs of fathers of preterm infants. The results may also contribute to developing guidelines and policies to promote psychological well-being and resilience among this population in the healthcare system.

**Ethics and dissemination:**

This study adheres to the International Ethical Guidelines for Biomedical Research and the Declaration of Helsinki. Approval has been obtained from the People’s Hospital of Deyang Human Research Ethics Committee (No: 2019-04-150-K01). The research follows the principles of open science, and the findings will be published while ensuring participants’ confidentiality.

## Introduction

Data from the World Health Organization (WHO) indicate that preterm births account for 10% of all births globally, with 15 million preterm babies born each year (1, 2). Of these,35% of infants die of due to various causes or complications, underscoring the significance of preterm birth as a global concern (3). China, as a populous country, has implemented various population policies, such as the one-child policy introduced in 1979, which was later adjusted to a two-child policy in 2015, and recently, a three-child policy in 2021. The three-child policy allows couples to have up to three children to address the aging population and declining birth rates (4). These policy changes have led to an increase in the average maternal age. Under the one-child policy, many families delayed having children until they felt financially stable, often resulting in older maternal ages at first birth. With the relaxation to a two-child and then a three-child policy, many families who had their first child later in life are now having additional children at even older ages. This shift may have implications for the occurrence of high-risk births and infant mortality, as older maternal age is associated with a higher risk of complications during pregnancy and childbirth. Data from the National Health Commission of the People’s Republic of China indicate that approximately 7.0% of births in China are preterm, making it a leading cause of infant mortality (5).

Preterm birth is an unexpected event that imposes significant stress and challenges on parents. Research has shown that it can lead to parental anxiety, depression, and even traumatic symptoms such as intrusive memories and attempts to avoid or ignore specific experiences and emotional triggers (6, 7). Fathers, as essential members of the family, play an indispensable role in the growth and development of preterm infants, and their psychological well-being significantly impacts the entire family. Additionally, fathers face physical and mental exhaustion as they balance their work responsibilities while providing emotional and financial support to the mother and family during the hospitalization and developmental process of preterm infants (8).

The mental health challenges faced by fathers of preterm infants have garnered increasing attention in recent research, which has primarily focused on identifying and quantifying negative psychological outcomes such as postpartum depression and anxiety. Studies have consistently found that postpartum depression in fathers of preterm infants occurs at rates 3 to 9 times higher than in fathers of full-term infants, and the adverse psychological impact of premature birth tends to persist longer, with heightened risks of psychological health issues observed up to nine months post-childbirth (9–13). Furthermore, it has been documented that these negative psychological experiences among fathers can adversely affect the cognitive and behavioral development of preterm infants, complicating the transition and development of family functioning (14, 15).

Post-traumatic growth (PTG) is a construct that was initially identified and defined and defined by Tedeschi et al, PTG is defined as the transformative positive changes in an individual’s cognition, emotions, and social functioning that can arise from coping with a traumatic event, influenced by demographic, event-related, and social factors (16–19). It is theorized that post-traumatic growth can manifest over five domains: self-perception, relating to others, new possibilities, appreciation of life, and existential change (20). There have been studies of post-traumatic growth in preterm parents. Anna et al. conducted a study on post-traumatic growth among parents following their infants’ admission to a neonatal intensive care unit (21). They found that post-traumatic growth was less influenced by sociodemographic variables or the stressors themselves, and was more significantly associated with psychological factors such as anxiety, depression, perceived vulnerability, and post-traumatic stress disorder. Wang et al. conducted a cross-sectional survey investigating post-traumatic growth and its influencing factors among parents of preterm infants (22). The findings revealed a moderate positive correlation between post-traumatic growth and variables such as perceived social support, rumination, and family resilience. Furthermore, multiple linear regression analysis identified purposeful contemplation, family resilience, educational level, family support, age, and marital status as significant influencing factors. Ghaedi-Heidari et al. implemented a three-week Mindfulness-Based Stress Reduction (MBSR) program for mothers of preterm infants in intensive care units (23). The results indicated that MBSR significantly enhanced Posttraumatic Growth (PTG) among these mothers. Consequently, it is recommended that this approach be incorporated into psychological support programs for mothers with premature infants in neonatal intensive care units.

These findings highlight the critical need to address the psychosocial challenges faced by parents after premature births. However, qualitative studies focusing on the post-traumatic growth (PTG) of fathers with preterm infants remain scarce. Recent studies have explored various aspects of posttraumatic growth (PTG) among parents of preterm infants. However, much of the existing research primarily focuses on mothers, leaving a significant gap in understanding the experiences of fathers. For instance, Ghaedi-Heidari and Aftyka examined PTG in mothers and parents but provided limited insights into the distinct challenges faced by fathers (23, 24). Furthermore, most research has been conducted in Western contexts, with few studies examining PTG among fathers in China. While mothers have traditionally been the primary focus in research on the care of premature infants, the role of fathers is equally crucial and often underrepresented, particularly in the context of Chinese culture. Traditional expectations of fathers as primary breadwinners are evolving, with more fathers participating in hands-on caregiving. This cultural shift makes it urgent to understand the unique experiences and challenges faced by fathers in this role, which has been underexplored in existing literature. Current research indicates that individuals who exhibit higher levels of PTG may experience reduced anxiety, depression, and other adverse psychological outcomes.

This study aims to build upon existing research by focusing on the underexplored area of post-traumatic growth among fathers of preterm infants. Recognizing the profound psychological distress these fathers may face, our research seeks to uncover how, despite these challenges, they can also experience significant personal growth and development. Exploring PTG in this context is essential not only for providing a more holistic understanding of fathers’ psychological experiences following preterm births but also for informing the development of supportive interventions that foster resilience and growth during this critical period.

## Methods and analyses

### Study design

This study protocol adheres to the Consolidated Criteria for Reporting Qualitative Research (COREQ) and Standards for Reporting Qualitative Research (SRQR), ensuring rigorous reporting standards for qualitative research (25). To explore the psychological processes fathers undergo following the premature birth of their child, this study will employ a phenomenological approach. Phenomenology is particularly suited for delving into the lived experiences of individuals, aiming to uncover the essence of these experiences through their own perspectives.

Data will be collected through individual semi-structured interviews, a method that aligns well with phenomenological research by facilitating in-depth exploration of participants’ practices, beliefs, opinions, and experiences. These interviews are designed to allow fathers the freedom to express their views in their own terms, capturing factual material and nuanced descriptions of their psychological processes.

Given the phenomenological nature of this study, we aim to conduct interviews with fathers of preterm infants. This sample size is informed by the principle of thematic saturation, where data collection continues until no new information or themes are observed. This estimated range is based on similar phenomenological studies, which suggest that saturation is typically achieved within this range of participants. However, we remain open to adjusting the number of interviews as needed to ensure a comprehensive understanding of the fathers’ experiences.

### Study participants

In this study, we aim to specifically explore the experiences and potential post-traumatic growth of fathers who have navigated the challenges associated with the preterm birth of their infants. The study aims to conduct interviews with 20-30 fathers or until theoretical data saturation is reached, ensuring that the data are sufficiently coherent and no new information emerges that could further address the study’s objectives. Should the data prove insufficient or fail to reach saturation, we will re-evaluate our recruitment strategies to include additional participants. Data saturation is defined as the point at which no new themes emerge from participant interviews (26–28). This may involve extending the recruitment period or broadening the criteria for inclusion to ensure a richer dataset. The inclusion criteria include as follows:

Inclusion Criteria:

- Fathers of preterm infants born with a gestational age of less than 37 weeks, aiming to cover a broad spectrum of prematurity and its associated challenges.
- The infant must have been discharged from the Neonatal Intensive Care Unit (NICU) at least six months prior to the study, allowing time for potential post-traumatic growth experiences to emerge, while ensuring that the challenges of early care are still within the reflective capacity of the fathers (29).
- The father should be the primary contact person during the hospitalization of the premature infant and actively involved in the ongoing care of the child, ensuring a direct understanding and involvement in the care process.
- Voluntary participation in the study, with informed consent provided.

Exclusion Criteria:

- Newborns with congenital malformations or hereditary diseases are excluded to focus the study on the experience of prematurity without the confounding factors of additional long-term care needs unrelated to prematurity.
- Fathers who have a diagnosed mental illness that would significantly hinder their ability to participate in the study or provide reliable responses.

### Sampling procedure

A purposive sample of fathers of preterm infants will be invited to participate in this study. Participants will be selected based on specific criteria to ensure a comprehensive understanding of their experiences. These criteria include being the biological father of an infant born prematurely (before 37 weeks of gestation) and having been involved in the care of their infant during the neonatal period.

Purposive sampling will be employed to identify fathers who can provide rich, detailed accounts of their experiences related to posttraumatic growth. This method ensures that the phenomenon of interest is explored through the perspectives of those most likely to offer valuable insights. Fathers who have navigated the challenges of caring for a preterm infant in the Chinese healthcare system during the specified period will be considered.

To represent a diverse range of experiences, efforts will be made to recruit participants from different regions across China. This approach will help capture a comprehensive picture of posttraumatic growth among fathers of preterm infants in varied geographical and cultural contexts within China.

### Interview setting

The interviews will be conducted in a designated meeting room at the Department of Pediatrics, People’s Hospital of Deyang City. This setting has been selected to ensure privacy and comfort, which are essential for facilitating an open dialogue and allowing fathers of preterm infants to share their experiences freely and without interruption. The room provides a controlled environment that is quiet and free from distractions, supporting a professional and supportive atmosphere. This arrangement also facilitates the establishment of rapport and trust, which are crucial for eliciting honest and comprehensive responses from the participants.

By focusing exclusively on in-person interviews, we aim to maintain consistency in the interview conditions and ensure the quality and reliability of the data collected.

### Recruitment strategies and data collection methods

Initially, all eligible participants will be contacted via telephone to invite them to take part in the study. Subsequently, they will receive more detailed information about the study’s aims and objectives, a consent form that needs to be signed during the meeting, and terms of participation via email or a messaging app to ensure an informed decision-making process. Once the participants have agreed to participate, individual appointments will be scheduled at a convenient date and time. If recruitment does not meet the anticipated levels, we will implement alternative strategies, such as reaching out to additional NICU centers or utilizing social media platforms aimed at parents of preterm infants, to enhance our participant pool.

Data collection will primarily be carried out via in-depth, semi-structured interviews conducted face-to-face. Each interview will be audio-recorded using a dictaphone or telephone and subsequently transcribed verbatim. To ensure the accuracy of the data and maintain transparency in our research process, transcripts will be shared with participants for their comments and/or corrections. This step, known as member checking, aims to enhance the credibility and validity of our research by allowing participants to verify the accuracy and authenticity of their conveyed experiences. Transcripts will be returned to participants within one week following the interview, and they will be given up to two weeks to provide feedback.

To minimize bias, interviews will be conducted by a qualified psychologist who is not a member of the core research team but is experienced in qualitative research methodologies and has extensive experience working in the Department of Neonatology. This psychologist, being of the same nationality as the participants (Chinese), the interviewer will maintain a sensitive and open-ended approach to questioning. Their external status helps to mitigate potential biases that might arise from the research team’s expectations or hypotheses. Participants will be reassured that the researchers are interested in their authentic experiences, feelings, and views, emphasizing that there are no “correct” answers.

At the conclusion of each interview, participants will be given the opportunity to share any additional insights about their experiences. The member checking procedure will also be employed to ensure that the final report accurately reflects the fathers’ authentic views and experiences. The report will include main themes and illustrative quotes.

A separate interview guide has been developed for the study, drawing from existing literature and the practical expertise of the lead author and an infant’s specialist (**Supplementary File S1**). The questions are predominantly open-ended and cover various subject areas. Each question includes supplementary prompts visible only to the interviewer, intended to provide further support or explanation if needed during the participants’ responses. The interview script will serve as a guide, but the participants will ultimately steer the direction of each interview.

Prior to the actual study, the interview guide will be pilot-tested with a sample father of a premature baby who will not be part of the main study but shares characteristics with the study participants. This pilot testing will enhance the accuracy of data collection, and the interview guide will then be re-evaluated and reviewed by the research team to make any necessary adjustments.

Each interview will begin with preliminary questions aimed at building rapport with the interviewee, leading to an in-depth discussion based on the open questions in the interview guide. After each interview, participants will be asked to complete a data sheet providing sociodemographic information.

In addition to the interviews and transcriptions, field notes will be taken to record observations on the interviewee’s overall demeanor, including nonverbal cues, bodily and facial expressions, gestures, and any speech alterations (e.g., laughter or crying).

Each interview is expected to last between 40 to 60 minutes.

To address the potential negative consequences of investigating difficult experiences, we will take several steps to ensure the well-being of the fathers participating in our study. Recognizing that discussing traumatic experiences can evoke strong emotions, including deep sadness, we will arrange for specialists in psychosomatic medicine and mental health (e.g., Liu Minghui and Huang Dandan) to provide ongoing support and communication with participants at several time points after the interviews (1-week, 1-month, 3-month, and 6-month). This continuous support aims to monitor and address any potential adverse effects caused by recalling distressing memories.

### Data analysis

Thematic analysis, as an independent qualitative description method, is primarily focused on identifying, analyzing, and reporting data patterns (30). In this study, thematic analysis will be employed, involving multiple listenings to each recorded interview to gain a comprehensive understanding of the data and the interviews as a whole (31). The analytical process will be guided by the interpretive description method and will be conducted in the following three stages:

Phase 1 Transcription: Audio files will be transcribed verbatim by the moderator (LNY), ensuring data integrity. Transcriptions will be meticulously cleaned and organized using Microsoft Word, preparing them for analysis.

Phase 2 Familiarization and Initial Coding: Both authors (DDH, YLL) will immerse themselves in the data by reading the transcripts multiple times. This immersion aids in familiarization with the content and initiates the process of generating initial codes.

Phase 3 Theme Identification: Both authors (DDH, YLL) will immerse themselves in the data by reading the transcripts multiple times. This immersion aids in familiarization with the content and initiates the process of generating initial codes.

In the first phase, qualitative data will be transcribed verbatim, carefully cleaned, and managed by the moderator (LNY). Microsoft Word will be utilized to facilitate the analysis process. In the second stage, both authors (DDH, YLL) will thoroughly read the entire transcript, familiarize themselves with its content, and begin the initial coding process (32). During the third stage, the two authors (DDH, YLL) will code and organize each interview based on the research questions, with assistance from NVivo V.10 software. Concurrently, constant comparison analysis will be employed to identify similar and different themes both within and across interviews. To enhance the credibility of the data, all authors will review the data multiple times for clarification and consensus until the explanatory themes are fully described. These steps will contribute to achieving a comprehensive insight into the data and providing a coherent and relevant description of the findings.

To address potential differences in opinions among researchers during theme extraction, we will adopt a consensus approach. This involves discussion and agreement on themes through regular team meetings, ensuring that all interpretations are thoroughly debated and agreed upon. If necessary, an external qualitative researcher may be consulted to provide an impartial perspective. NVivo V.10 software will be utilized to assist in organizing, coding, and identifying themes within the data. This tool supports a systematic approach to qualitative data analysis, facilitating the management of large datasets and enhancing the reliability of our findings. In the process of data analysis, we will ensure rigor by considering various cases in different situations. The study will distinguish between premature infants who are considered high-risk due to additional health issues (such as heart conditions requiring surgeries or frequent medical interventions) and those without such complications. This distinction will allow for a nuanced analysis of the fathers’ experiences, recognizing that the intensity and nature of challenges can vary significantly. To accurately reflect the varied experiences of fathers, the study will categorize participants based on the gestational age of the infant at birth (e.g., extremely preterm: less than 28 weeks, very preterm: 28 to <32 weeks, and moderate to late preterm: 32 to <37 weeks). This categorization will help in understanding the differential impacts of prematurity levels on fathers’ experiences and potential for post-traumatic growth.

### Patient and public involvement

Because this protocol is a public health initiative, there will be no patient involvement. This study aims to engage fathers of premature infants as participants. Participants will not be involved in the design of the study, the development of interview questions, or the conduct of the study. They will, also, be invited to comment on the transcripts and results of the analysis. A summary of the findings will be shared with those interested.

## Discussion

The aim of this study is to delve into the posttraumatic growth experienced by fathers of preterm infants during their journey of caring for their premature babies. By utilizing qualitative research methods, we strive to gain a profound understanding of fathers’ experiences and psychological transformations throughout this process, as well as the impact of these experiences on their personal growth and adaptation. By discussing the purpose and methodology of this study, we aim to identify potential mechanisms and develop possible interventions to support posttraumatic growth among fathers of preterm infants.

By analyzing the caregiving experiences of fathers, we anticipate uncovering themes and patterns such as stress and anxiety, concerns about their child’s health, and reevaluation of their roles as fathers. These themes will facilitate a deeper comprehension of fathers’ psychological responses and adaptive processes when confronted with adversity and challenges.

Exploring the process of posttraumatic growth among fathers of preterm infants holds significant implications for practice and policy. Firstly, the research findings can offer valuable insights to healthcare professionals, enabling them to better support the psychological well-being and posttraumatic growth of fathers in this context. Secondly, the results can inform the development of relevant interventions aimed at promoting positive adaptation and psychological growth among fathers of preterm infants (33).

However, while qualitative research methods provide the advantage of exploring individual experiences, certain limitations must be acknowledged. Firstly, sample selection may be constrained by factors such as geographical location and cultural backgrounds, which may affect the generalizability of the research findings. Secondly, addressing and controlling subjectivity and researcher bias during the study process are essential considerations.

For future research, we recommend employing multiple methods, such as mixed methods, to validate and expand upon the findings of qualitative research, thus enhancing the reliability and generalizability of the research. Additionally, recruiting a more diverse sample and conducting cross-cultural comparisons in various cultural and societal contexts can provide a more comprehensive understanding of posttraumatic growth. Specifically, future studies could explore longitudinal designs to track the evolution of posttraumatic growth over time, and quantitative methods to measure the impact of specific interventions designed to support fathers. Moreover, investigating the role of additional variables such as socioeconomic status, education level, and access to healthcare services could provide deeper insights into the factors influencing posttraumatic growth.

In conclusion, this study protocol presents a framework for qualitative research aimed at exploring the phenomenon of posttraumatic growth among fathers of preterm infants. By gaining an in-depth understanding of their experiences and psychological changes during the journey of caring for premature infants, we can offer new insights for improving support and interventions for fathers in this specific context. Future research can further validate and expand upon these findings, contributing to a comprehensive understanding of posttraumatic growth among fathers of preterm infants.

### Strengths and limitations

The qualitative approach of this study allows for a comprehensive exploration of fathers’ experiences and growth following premature birth, providing rich insights into their psychological transformations. By collecting detailed and nuanced data, we will gain a thorough understanding of the unique challenges and coping mechanisms of fathers of premature infants. This in-depth exploration will offer valuable information that can inform targeted interventions and support systems.

However, the study’s generalizability is limited by several factors. Firstly, the small number of participants may not represent the broader population of fathers of preterm infants. Additionally, cultural and social factors specific to the study’s context might affect the broader applicability of our findings to different populations of fathers. Participants’ self-reporting may also be influenced by social desirability or recall bias, potentially impacting the accuracy of the data collected. Furthermore, conducting the research at a single location limits the diversity of experiences and perspectives captured, which may affect the generalizability of the findings to other contexts. These limitations highlight the need for future research to employ mixed methods, recruit more diverse samples, and conduct cross-cultural comparisons to enhance the reliability and applicability of the findings.

### Ethics and dissemination

This study complies with the International Ethical Guidelines for Biomedical Research and the Declaration of Helsinki, having been approved by the People’s Hospital of Deyang Human Research Ethics Committee (Approval No: 2022-04-150-K01), as detailed in **Supplementary File S2**. The research team will execute the study following all pertinent guidelines and regulations. Prior to conducting interviews, written informed consent will be secured from all participants, who will be thoroughly briefed about the study’s aims and the voluntary basis of their involvement. To safeguard participant anonymity and confidentiality, all personal identifiers will be removed during data analysis and reporting.

The research team is dedicated to the widespread dissemination of the project’s findings through various channels, including academic publications, conference presentations, workshops, and webinars, aiming to contribute valuable insights to the wider scientific and medical communities. The results of this study will be shared at relevant conferences, published in peer-reviewed journals, and disseminated among all interested parties.
