# Supplementary material for: The posttraumatic growth of fathers of preterm infants: protocol for a qualitative study in China
Source: Front Psychiatry. 2024 Aug 30;15:1444226. doi: 10.3389/fpsyt.2024.1444226 (PMC11393778; doi:10.3389/fpsyt.2024.1444226)
Supplement: Supplementary file 1 [file DataSheet1.pdf]

Interview guide

•Introduce yourself and explain the purpose of the study.

•Establish rapport and create a comfortable atmosphere.

•Seek consent for audio recording or note-taking during the interview.

1.Personal Background and Experience

•Ask about the participant's background information (e.g., age, occupation, education).

•Explore their journey of becoming a father of a premature infant and their initial reactions and emotions.

•Understand their understanding of and expectations for the role of a father of a premature infant.

2. Premature Infant Care Experience

•Inquire about the participant's specific experiences and emotions during their premature infant's hospitalization.

•Explore their interactions and support from the healthcare team.

•Understand the challenges they faced during the premature infant care process and their coping strategies.

3.Father's Mental Health and Growth

•Explore the participant's mental health status during the premature infant experience, including anxiety, depression, or other emotions.

•Inquire about any personal growth or positive changes they may have experienced and explore related experiences and influences.

•Understand their perspectives on support and interventions for fathers in similar situations.

4.Family and Social Support

•Inquire about the participant's support system and resources within their family and community.

•Explore their communication and support with their spouse, family members, and the broader social network.

•Gather their suggestions and recommendations for improving family and social support for fathers of premature infants.

5.Closing and Wrap-up

- Thank the participant for their time and willingness to share their experiences.
- Confirm if there are any additional important points or experiences, they would like to add.
- Ask if they have any questions or if there's anything else they would like to discuss.
