# Supplementary material for: The posttraumatic growth of fathers of preterm infants: protocol for a qualitative study in China
Source: Front Psychiatry. 2024 Aug 30;15:1444226. doi: 10.3389/fpsyt.2024.1444226 (PMC11393778; doi:10.3389/fpsyt.2024.1444226)
Supplement: Supplementary file 2 [file DataSheet2.pdf]

德阳市人民医院
DEYANG PEOPLE'S HOSPITAL

The Medical Ethics Committee reviews the approval

（YXL-OP-007-A06-V2.0）

Ethics approval of People's Hospital of Deyang Human Research Ethics Committee

Ethical Review No.**(2022-04-150-K01)（V2.0）**

Project Title	**A Study on the Psychological Health of Fathers of Preterm Infants**	
Method of review	□Regular meeting review □Emergency meeting review ☑Rapid review	
Venue	Ethics Committee Office of People's Hospital of Deyang	
Research Unite	People’s Hospital of Deyang	
Department	Department of Nursing	Principal investigators: Kejimu Sunzi, Cheng Lei
Content of review	1. Application for Initial Review of Medical Ethics;<br>2. Research Program (version number, date);<br>3. Informed consent;<br>4. Resume of Researcher.	

Review Result	Approved		
Frequency of continuous review	☐ Every3 Months ☐ Every 6 Months ☑ Every12 Months □Not applicable		
Validity of approval	20/2/2022-20/2/2024		
Contacts	Wei Zhou	Tel	0838-2312773<br>0838-2418826
Signature of Chairman	Wei Zhou		
Date	20/2/2022	Ethics Committee（stamp）	
Address	No.173 Taishan Road, Jingyang District, Sichuan Province, China		

Note

1.According to the ethical principles of the Measures for the Ethical Review of Biomedical Research Involving Human Beings and the Helsinki Declaration, the Ethics Committee has reviewed and agreed researchers to carry out this research according to the approved documents.

2.Please conduct the study in accordance with relevant laws and regulations and the protocol approved by the Ethics Committee to protect the health and rights of the subjects.

3. After the study is started, the Ethics Committee will conduct regular follow-up review every 12 months. Please submit the research progress report/follow-up review report one month in advance. The Ethics Committee has the right to change the follow-up review frequency according to the actual development of the study.
